# Minimally invasive, ultrasound-guided synovial tissue biopsies are well-tolerated, safe and feasible in children and young people with inflammatory arthritis

**DOI:** 10.1136/rmdopen-2026-006817

**Published:** 2026-10-14

**Authors:** Elizabeth Clay, Chrissy Bolton, Annie Hackland, Charlotte G Smith, Klaudia Kupiec, James Kelly, Samantha Chippington, Zishan Sheikh, Charlene Foley, Patricia Reis Nisa, Søren Lomholt, Vicky Alexiou, Inga Turtsevich, Chadwick Pils, Sugrah Sultan, Neelam Khan, Manigandan Thyagarajan, Georgiana Neag, Christopher B Mahony, Holly R Adams, Catherine Cotter, Miriam Sadler, Huong Nguyen, Ece Beren Barklam, Luke J Jones, Paulynn Suyin Chin, Samuel Kemble, Lucy-Jayne Marsh, Penny Davis, Sunit Davda, Sandrine Compeyrot-Lacassagne, Fahim Patel, Elizabeth C Rosser, Megan M Hanlon, Will Coles, Edward Dicarlo, Ellen Gravallese, Andrew Filer, Eslam Al-Abadi, Lucy R Wedderburn, Adam P Croft

**Affiliations:** 1 School of Infection Inflammation and Immunology, College of Medicine and Health, University of Birmingham, Birmingham, UK; 2 Kennedy Institute of Rheumatology, University of Oxford, Oxford, UK; 3 National Institute for Health and Care Research (NIHR), Biomedical Research Centre at Great Ormond Street Hospital NHS Foundation Trust, London, UK; 4 Infection, Immunity and Inflammation Research and Teaching Department, University College London Great Ormond Street Institute of Child Health, London, UK; 5 Centre for Adolescent Rheumatology at UCL, UCL Hospitals and Great Ormond Street Hospital (GOSH), London, UK; 6 Department of Rheumatology, Great Ormond Street Hospital NHS Foundation Trust, London, UK; 7 Department of Interventional Radiology, Great Ormond Street Hospital NHS Foundation Trust, London, UK; 8 Birmingham Women’s and Children’s Hospital NHS Foundation Trust, Birmingham, UK; 9 Department of Biomedicine, Aarhus University, Aarhus, Denmark; 10 Department of Pediatric and Adolescent Medicine, Aarhus University Hospital, Aarhus, Denmark; 11 Department of Ageing, Rheumatology and Regenerative Medicine, Division of Medicine, UCL, London, UK; 12 Division of Rheumatology, Inflammation, and Immunity, Department of Medicine, Brigham and Women's Hospital and Harvard Medical School, Boston, Massachusetts, USA; 13 Department of Pathology and Laboratory Medicine, Hospital for Special Surgery, New York, New York, USA; 14 National Institute for Health and Care Research (NIHR) Biomedical Research Centre: Birmingham, UK, Birmingham, UK

**Keywords:** Arthritis, Arthritis, Juvenile, Child, Synovitis

## Abstract

**Objective:**

To evaluate feasibility, tolerability and acceptability of performing minimally invasive synovial tissue biopsies for the purpose of research in children and young people diagnosed with inflammatory arthritis.

**Methods:**

Synovial tissue biopsies were obtained from the joints of children with arthritis undergoing intra-articular procedures performed under general anaesthetic, and embedded within routine clinical care pathways. Questionnaires pertaining to the tolerability of the procedure were completed. Tissue samples were processed for histological assessment of tissue quality and graded for synovitis.

**Results:**

Synovial tissue biopsies were obtained from 62 children, aged between 1 and 17 years of age (median 7.1 years). While most children had a single joint biopsied, seven procedures involved biopsy of two different joints. At baseline, 65 knees, two ankles and two wrist joints were biopsied; seven children underwent repeat knee biopsies later in their disease. Almost 63% of biopsies (n=39/62 participants) were performed in children with oligoarticular juvenile idiopathic arthritis (JIA). Postprocedure questionnaires revealed a majority of families (80%, n=32/40) would be ‘very likely’ or ‘somewhat likely’ to consent to a repeat biopsy in the future. Histological assessment of tissue using an adapted Krenn synovitis score confirmed differences between oligoarticular and polyarticular JIA. No serious adverse events were reported.

**Conclusions:**

Ultrasound-guided synovial tissue biopsies in children undergoing intra-articular procedures under general anaesthetic are feasible, safe and well-tolerated, and can be embedded within routine clinical care pathways for the procurement of tissue samples for the purpose of research.

**WHAT IS ALREADY KNOWN ON THIS TOPIC**

- Synovial tissue research in adults with inflammatory arthritis has led to the identification of new potential biomarkers to guide treatment selection and the exploration of novel treatment targets.
- Synovial tissue research in children has been lacking due to a paucity of data on the feasibility, tolerability and safety of synovial tissue biopsy procedures in younger children and in the early phases of arthritis.

**WHAT THIS STUDY ADDS**

- Ultrasound-guided synovial tissue biopsies performed under general anaesthetic during routine joint injection procedures are safe, well-tolerated and acceptable to children, young people and their families.
- Synovial tissue samples acquired during routine joint injection procedures are of high quality and can be used for the purpose of research.

**HOW THIS STUDY MIGHT AFFECT RESEARCH, PRACTICE OR POLICY**

- This study provides a framework for performing synovial tissue research in children and young people with inflammatory arthritis that will allow us to address the urgent unmet need to define synovial tissue pathology in the early phases of juvenile idiopathic arthritis.

## Introduction

 Juvenile idiopathic arthritis (JIA) is the most common inflammatory rheumatic disease of childhood, manifesting as persistent joint inflammation in children/young people (CYP) aged 16 years of age and under.^1^ Currently, there are seven subtypes of JIA defined by the International League of Associations for Rheumatology (ILAR) classification criteria, which provides a framework for conducting clinical research using a common nomenclature.^2^ However, the ILAR classification is based largely on clinical presentation, and a lack of molecular tissue pathotyping means that, with few exceptions, the classification criteria do not inform treatment selection or predict drug response.^3 4^ To align pathogenic mechanisms to treatment selection, the cellular and molecular pathways underpinning disease heterogeneity and variation in response to specific targeted therapies must be defined. This will facilitate development of precision, evidence-based treatment stratification approaches to improve long-term outcomes for CYP affected by arthritis.^4^

Over the last decade, research in adult-onset inflammatory arthritis has shifted the focus to synovial tissue pathotyping as a means of defining disease heterogeneity that may underpin treatment response.^5–9^ These studies have transformed our understanding of the cellular and molecular basis of joint inflammation,^5 7 8^ leading to the identification of molecular tissue signatures that align with response to drug treatment in otherwise clinically indistinguishable individuals.^6 7 9 10^ Such studies have been facilitated by the application of ultrasound-guided, minimally invasive joint biopsy techniques, which allow investigators to obtain synovial tissue samples across the life-course of disease, including at the very earliest phases of disease onset, or before and after treatment perturbations.^7 10 11^ Furthermore, advances in tissue analytics, including single cell profiling technologies, have enabled target tissue analysis at molecular resolution.^6 12^ As a community, we now have the tools to define synovial tissue pathology and exploit its potential to improve the long-term outcomes for people with inflammatory arthritis.^13^

Despite the transformative potential of synovial tissue research for improving our understanding of the pathophysiology of JIA, there remains a reluctance to perform synovial tissue biopsies in CYP, in part due to a paucity of data on their safety and acceptability to families. This has led to a lack of understanding of synovial tissue pathobiology in JIA and a reliance on blood and synovial fluid analysis as surrogates of target tissue pathology.^3^ We have established a multicentre observational cohort study, in which CYP diagnosed with JIA underwent minimally invasive synovial tissue biopsies during routine joint injection procedures performed for the treatment of their arthritis, as recently highlighted.^13^ Here, we report on the safety, feasibility and tolerability of 79 ultrasound-guided synovial tissue biopsy procedures performed in 62 CYP with JIA.

## Methods

### Study cohort and design

Participants were aged 17 years and under, had a clinical diagnosis of JIA with onset before the age of 16 years and active arthritis on clinical examination at the time of procedure. Treatment-naïve status was defined according to those who had not received oral or intra-articular steroid, disease-modifying antirheumatic drug (DMARD) or biologic treatment at the time of biopsy. Needle biopsies were performed at two UK sites — Birmingham Women’s and Children’s NHS Foundation Trust (BWC) and Great Ormond Street Hospital (GOSH) — embedded in routine clinical care linked to intra-articular procedures. Baseline and follow-up clinical data including, but not limited to, patient demographics, clinical disease activity measures, repeat biopsy procedures, medication history and presence of uveitis were collected until July 2025 and recorded in the study database. Disease severity was scored using clinical Juvenile Arthritis Disease Activity Score 10 (cJADAS10 score), comprising the physician’s visual analogue scale (VAS), patient’s VAS and active joint count (maximum joint count censored at 10^14^).

### Patient and public involvement

CYP and families affected by JIA were involved at every stage of this research, including study conception, design, ethical approval and delivery, with input from our patient partnership stakeholder group, the CLUSTER Champions (https://www.clusterconsortium.org.uk/) and the UK Tissue Research in Childhood-Onset Inflammatory Arthritis (TRICIA) Consortium (https://tricia.network). Ongoing involvement has included specific events bringing families and researchers together to disseminate findings and receive feedback and guidance from patients and their families.

### Ultrasound-guided synovial tissue biopsies and ultrasound grading

Ultrasound evaluation was performed using GE HealthCare Logiq E9 and E10 (GOSH) and GE HealthCare Logiq S8 and Samsung V8 systems (BWC). Ultrasound evaluation and biopsies were performed by experienced interventional radiologists at each site. Merit Medical Temno Evolution coaxial biopsy devices were used to obtain samples. Biopsies were only performed in joints where the synovial membrane could be visualised by ultrasound scan. Synovial hyperplasia was assessed with ultrasound grayscale, and vascularity was graded with power Doppler, using a severity score of 0–3 according to defined criteria.^15^ Biopsies were only performed when sufficient synovial hyperplasia could be identified by the operator.

All CYP received general anaesthetic during the procedure. For knee biopsies, CYP were positioned supine with knees flexed up to 30° using supportive pads; in some cases, extension of the knee was necessary to prevent synovial fluid being forced out of the viewing plane. The procedure was performed under strict sterile conditions, including establishment of an aseptic field using betadine or chlorhexidine skin disinfection and appropriate surgical drapes. Operators used high frequency 6–15 MHz linear probes optimised for paediatric musculoskeletal ultrasound to identify synovial hypertrophy in the suprapatellar recess.

Local anaesthesia was achieved with injection of 5–10 mL of 1% lignocaine or 2.5 mg/mL levobupivacaine into skin, subcutaneous tissues and the joint capsule. Following local anaesthesia, a co-axial introducer needle was inserted into the joint. Synovial fluid was aspirated via the co-axial needle, as part of the therapeutic procedure. Following synovial fluid aspiration, a 14–16 G biopsy needle was advanced to the synovial space via the introducer under ultrasound guidance. Synovium was sampled from the medial or lateral recess depending on the area of greatest synovial thickening and vascularity. Samples were taken with the biopsy needle from targeted areas of synovial hypertrophy to obtain approximately 6×10 mm cores from each joint. At the operator’s discretion, a further 5–10 mL of 1% lignocaine or 2.5 mg/mL levobupivacaine and 5–10 mL of normal saline was introduced into the joint to help separate synovial folds so that the inner lining layer could be targeted for biopsy. In the event of significant haemarthrosis during biopsy, 20–100 mL of normal saline was used to washout blood products from the joint. Following biopsy, if clinically indicated, corticosteroid was injected into the joint via the in situ co-axial introducer and the skin incision site was compressed prior to application of 2-Octyly Cyanoacrylate (Dermabond) or a sterile dressing.

### Sample storage and preprocessing

Biopsy cores of synovial tissue were dissected into approximately three equal fragments and split between downstream analytical platforms to give an overall representation of the synovium. Fragments deemed excessively blood-stained were discarded. Formalin-fixed tissue samples were embedded in paraffin within 48 hours and processed for embedding using Leica Polaris II (processor) and Solmedia MPS/P2 (embedder), or Leica TP1020 (processor) and Leica Histocore Arcadia H (embedder). Synovial tissue histology and grading, immunohistochemistry and immunofluorescence were performed as per online supplemental Table 1.

### Preprocedure and postprocedure questionnaires

To assess the tolerability of synovial biopsies in CYP, two sets of questionnaires were completed by the parents of study participants, or the participants if they were able to complete the questionnaire themselves. First, VAS scores (0–100 mm) regarding pain, stiffness and swelling of the biopsied joint were collected both before (‘pre’) and after (‘post’) the procedure; ‘pre’ VAS scores were completed on the day of the biopsy, except two that were completed the day before.

Second, tolerability questionnaires were completed during a follow-up phone call postprocedure using a 5-point Likert scale. The following questions were included: *immediately after the procedure and the following day, thinking only about the joint from which samples were taken:*

- *How much pain/discomfort did you/your child experience in the joint that was biopsied?*
- *Compared with before the procedure, how do you think the joint which was biopsied now feels?*
- *If asked to undergo/have your child undergo the same procedure on the same or another joint, how likely would you be to say yes?*

Responses completed within 30 days of the procedure only were included in the analysis.

### Data storage, analysis and statistical approach

Study data were collected and managed using the Research Electronic Data Capture electronic database hosted at the University of Birmingham, and collection of follow-up data continued postprocedure. Patient data collection is terminated on request from the patient or when the patient transitions out of the trust or to adult care. For adverse events, a reporting system was established to categorise severity and define causality (online supplemental table 2). Data were analysed and statistics calculated using RStudio (V4.3.1). Shapiro-Wilk tests were used to assess normality, and variance assessed where necessary. For correlation analysis, Spearman’s rank correlation analysis was subsequently applied. Further statistical analyses were performed as follows: the Kruskal-Wallis test was used to compare three or more groups; the Wilcoxon signed-rank test was used for both paired and unpaired samples that were not normally distributed; Student’s t-test was used for unpaired samples with a normal distribution and Welch’s two-sample t-test was used for unpaired samples with unequal variances. 95% CIs were calculated. For postprocedure tolerability Likert scale questionnaires, statistical analysis was performed using one-way and 3×5 contingency table χ^2^ tests. Bonferroni correction was applied to p values when multiple comparisons were made.

## Results

### Participant details

Participants were invited to participate in the study following a clinical diagnosis of JIA and if undergoing an intra-articular procedure (such as intra-articular steroid injection or synovial fluid aspiration) as part of their clinical care or an alternative procedure requiring general anaesthetic (figure 1A–C). Joints were evaluated by ultrasound imaging, and the operator proceeded to biopsy only after confirmation of visible synovium (figure 1C). In only one child (aged 1.9 years, with two inflamed knee joints) was a biopsy attempted following ultrasound imaging, but no tissue ultimately retrieved. This child was, therefore, excluded from the cohort data.

**Figure 1:**
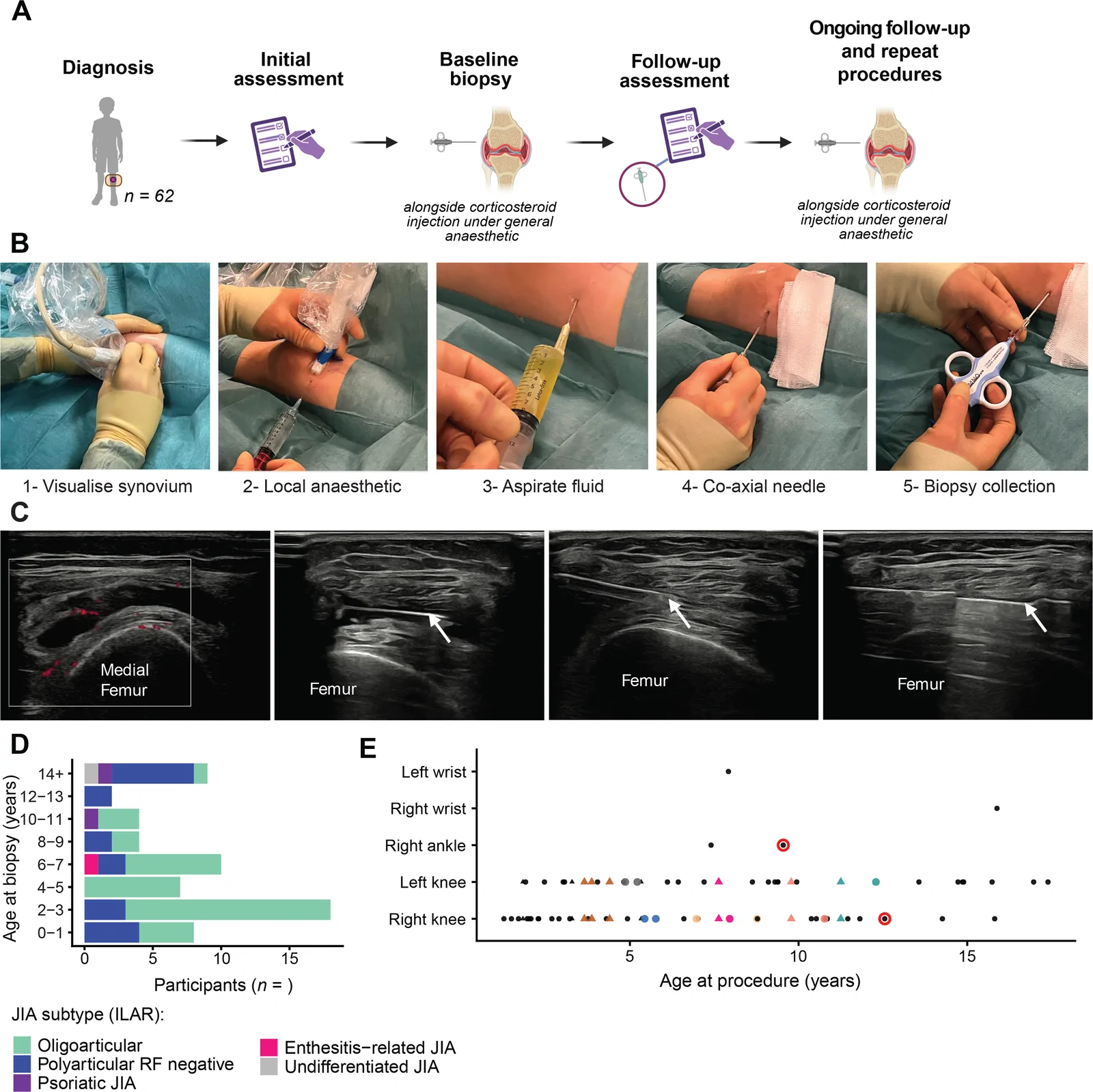
Performing minimally invasive synovial tissue biopsies in children. (**A**) Schematic of the study design. Prior to the biopsy procedure, pain, stiffness and swelling were assessed using visual analogue scales. Biopsy was performed during intra-articular corticosteroid injection procedure under general anaesthetic. An initial follow-up was carried out to assess joint symptoms and biopsy tolerability. Further follow-up was recorded, including additional joint procedures. (**B**) Sequential photographs of ultrasound-guided synovial fluid and tissue sampling from the inflamed knee joint of a child with JIA. (**C**) Representative examples of ultrasound images from the knee joint of a child with JIA during biopsy procedure. First: example of power Doppler (red); second: needle introduced into the joint; third: inner stylet removed, leaving the hollow co-axial needle chamber; fourth: biopsy needle introduced via the co-axial needle, with the cutting notch exposed. White arrow indicates the location of the needle in the joint during sample collection. (**D**) Bar plot showing age at clinical diagnosis, coloured by diagnosis (ILAR category), for those undergoing biopsy. (**E**) Plot showing joint biopsied (y-axis) by age at biopsy (x-axis) for a total n=79 biopsies, including baseline (n=69) and repeat (n=10) biopsies. Matching coloured symbols refer to the same patient, highlighting repeat biopsies. Triangles indicate biopsies where two joints were biopsied in one procedure, whereas circles indicate procedures where one joint was biopsied. Red-circled points highlight procedures where a concomitant steroid injection did not occur. Infographic in (**A**) created using https://BioRender.com and Adobe Illustrator. ILAR, International League of Associations for Rheumatology; JIA, juvenile idiopathic arthritis; RF, rheumatoid factor.

During the study period, synovial tissue biopsy procedures were performed in 62 CYP with arthritis, aged between one and 17 years (median 7.1, IQR 3.2–10.7; table 1, figure 1D,E). Consistent with other European observational study cohorts of individuals with non-systemic JIA,^16 17^ there was a female preponderance (74.2%, n=46/62) and most participants were of white ethnicity (71.0%, n=44/62), with other ethnic groups well-represented within the cohort (table 1). The ethnic diversity of CYP recruited to the study closely matched that published in the 2021 UK Government Census data for England and Wales.^18^

**Table 1:** Participant demographics and disease characteristics of the study cohort at baseline

Participant demographics (n=62)	
Age on day of procedure in years, median (IQR)	7.1 (3.2–10.7)
Female biological sex, n (%)	46 (74.2%)
Ethnicity, n (%)	
White	44 (71.0%)
Asian	9 (14.5%)
African	2 (3.2%)
Mixed	3 (4.8%)
Other/Not stated	4 (6.5%)
Disease characteristics at study entry	
Age at clinical diagnosis in years, median (IQR)	5.3 (2.5–9.4)
Diagnosis, ILAR subtype, n (%)	
Oligoarticular	39 (62.9%)*
Polyarticular RF negative	19 (30.6%)
Psoriatic JIA	2 (3.2%)
Enthesitis-related JIA	1 (1.6%)
Undifferentiated JIA	1 (1.6%)
Uveitis ever recorded during study period, n (%)	
Uveitis	14 (22.6%)
No uveitis	48 (77.4%)
cJADAS10 (total n=58), median (IQR)	
All JIA	9.1 (5.9–13.9)
Oligoarticular (n=37, n=2 NR)	7.1 (4.8–9.7)
Polyarticular RF negative (n=17, n=2 NR)	14.5 (12.4–19.4)†
Active joints at baseline (total n=61), mean±SD	
Oligoarticular (n=39)	1.9+1.2
Polyarticular RF negative (n=18, n=1 NR)	9.8+8.7
Psoriatic JIA (n=2)	9+5.7
Enthesitis-related JIA (n=1)	2
Undifferentiated JIA (n=1)	7
Childhood Health Assessment Questionnaire score (total n=62), median (IQR)	1 (0.4–1.6)
Physicians VAS (total n=58), median (IQR)	2.5 (1.5–3.5)
ANA status (total n=60), n (%)	
Positive	48 (80%)
Negative	12 (20%)
RF antibody status (total n=55), n (%)	
Positive	3 (5.5%)
Negative	52 (94.5%)
ESR at baseline (total n=55), median (IQR)	22 (12.5–49)‡
Treatment prior to biopsy procedure (total n=18, 29% of cohort)	
Joint injection alone	9
Oral steroid alone	0
DMARD alone (*being used at the time of biopsy*)	3 (*2*)
Joint injection and DMARD	3
Joint injection and oral steroid	1
DMARD and oral steroid (*being used at the time of biopsy*)	1 (*1*)
Joint injection, oral steroid and DMARD	1

N=62 unless otherwise stated.

* For two oligoarticular participants, disease had extended at point of entry to study.

† P=2.1×10−5, Welch’s two-sample unpaired t-test comparing cJADAS10 scores between oligoarticular and polyarticular RF-negative participants.

‡ ESR recorded nearest to biopsy date.

ANA, Anti-nuclear antibody; cJADAS10, clinical Juvenile Arthritis Disease Activity Score 10; DMARD, disease-modifying antirheumatic drug; ESR, erythrocyte sedimentation rate; ILAR, International League of Associations for Rheumatology; JIA, juvenile idiopathic arthritis; NR, not recorded; RF, rheumatoid factor; VAS, visual analogue scale.

Almost 63% (n=39/62) of participants had oligoarticular JIA (oJIA), while 31% (n=19/62) had polyarticular rheumatoid factor-negative JIA (RF-pJIA) (figure 1D, table 1). Most participants tested positive for antinuclear antibodies (ANA) (80%, n=48/60). In contrast, 5.5% (3/55) were rheumatoid factor (RF)-positive; of these, none had a polyarticular diagnosis. During the study period, 22.6% (n=14/62) of participants had a diagnosis of anterior uveitis. Participants had a median ESR of 22 at time of biopsy (IQR 12.5–49, n=55) and a median disease score of 9.1/30 (cJADAS10, IQR 5.9–13.9). Participants with RF-pJIA had a significantly higher cJADAS10 score to participants with oJIA (p<0.001, Welch’s two-sample unpaired t-test). Consistent with this, participants with RF-pJIA had a mean active joint count of 9.8 compared with 1.9 in those with oJIA.

The majority of participants (71%, n=44/62) were naive to treatment prior to baseline biopsy (table 1). Eight participants had received treatment with a DMARD prior to biopsy; of these, three participants were still taking DMARDs (all methotrexate) at the time of biopsy; one of these participants was also still taking oral steroids. Fourteen participants had received previous intra-articular steroid injections; however, none of these were within the 4 weeks prior to the biopsy procedure.

### Characteristics of minimally invasive synovial tissue biopsy procedures in CYP with arthritis

A median of six tissue cores were obtained per joint biopsy (IQR 6–8, online supplemental figure 1, table 2). When analysing only knee joints, there was a correlation between number of cores obtained during the procedure and the age of the participant at the time of the procedure (R=0.34, p=0.005, online supplemental figure 1A). There was, however, no correlation between the number of cores obtained and ultrasound greyscale score, which provides a measure of synovial tissue thickness (online supplemental figure 1B).

**Table 2:** Baseline biopsy details for n=62 participants (69 joints)

Days from diagnosis to biopsy (n=62), median (IQR)	24.5 (16–87.2)
Joints biopsied in baseline procedures (n=69)	
Right knee	35
Left knee	30
Right wrist	1
Left wrist	1
Right ankle	2
Number of tissue cores removed, median (IQR) (n=69 joints)	6 (6–8)

From baseline procedures performed in 62 participants, 69 joint biopsy samples were collected. Most participants (88.7%, n=55/62) had only one joint biopsied at baseline, while seven participants had two joints biopsied during the same procedure (triangles, figure 1E). At baseline, the following joints were biopsied: 65 knees, two ankles and two wrists (table 2 and figure 1E). Only two procedures involved a joint biopsy without a concomitant steroid injection (circled red, figure 1E). Ten joints were biopsied during repeat procedures in seven participants; all joints biopsied were previously sampled knee joints (coloured points; figure 1E). Therefore, in the study window, a total of 79 synovial tissue biopsies were collected. The analysis in this manuscript was performed primarily on baseline procedures.

### Synovial tissue biopsies are acceptable and well-tolerated in CYP with JIA

To assess the tolerability and acceptability of synovial tissue biopsies in paediatric cohorts, questionnaires regarding ‘pre’- and ‘post’-procedure symptom scores and biopsy tolerability were completed by participants or their parents, as appropriate, and results regarding baseline procedures were analysed (figure 2A–D). Questionnaires completed within 30 days of the procedure were included in the analysis (n=40). Thirteen participants completed questionnaires >30 days after the procedure and were, therefore, excluded from the analysis, while nine participants did not complete post-procedure questionnaires because attempts to contact them by telephone were unsuccessful. Clinical and demographic characteristics, including baseline diagnosis, did not differ significantly between these groups of participants (online supplemental figure 2 and online supplemental table 3).

**Figure 2:**
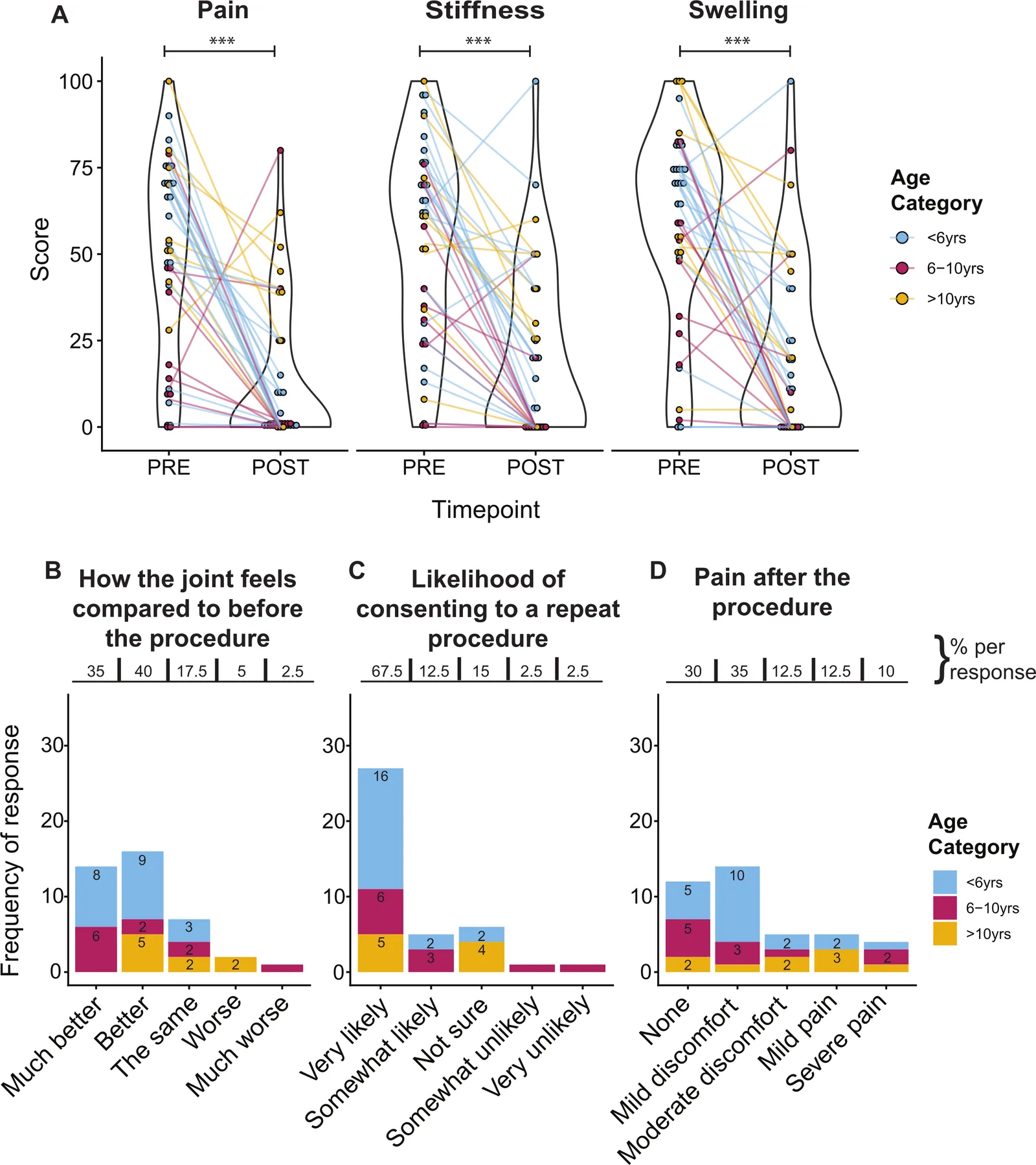
Tolerability of synovial tissue biopsies in children with juvenile idiopathic arthritis. Preprocedure and postprocedure tolerability was assessed. On each graph, the data are split by age category <6 years=blue; 6–10 years=maroon; >10 years=yellow. (**A**) Violin plots of questionnaire responses regarding joint pain, stiffness and swelling in the specific biopsied joint(s), completed before (PRE) and after (POST) the synovial biopsy procedure, using a visual analogue scale (VAS). Significance was assessed by Wilcoxon signed-rank tests: pain (V=45.5, p=4.04×10^−6^, 95% CI −50 to −27), stiffness (V=50.5, p=3.59×10^−6^, 95% CI −44.5 to −23.5) and swelling (V=32, p=2.34×10^−6^, 95% CI −54 to −31). ***, p < 0.001. (**B**) Follow-up Likert-scale responses regarding the biopsied joint(s) immediately after procedures and the next day. Question responses were analysed by comparing how the joint felt with how it felt before the procedure (**B**), the likelihood of agreeing to a repeat procedure (**C**) and pain postprocedure (**D**). Overall percentages for each response are listed at the top of each plot. One-way χ^2^ statistical tests were performed for each question, with all age groups combined (**B-D**) (**B**) χ^2^=23.25, df=4, p=0.0001; (**C**) χ^2^=59, df=4, p=4.7×10^–12^; (**D**) χ^2^=10.75, df=4, p=0.03. For (**A-D**), analysis was limited to VAS and questionnaires completed within 30 days after baseline procedures ((**A**) n=39; (**B–D**) n=40), and Bonferroni’s post hoc test used to adjust for three independent hypotheses (p_adj_<0.017).

Participants and their families recorded significantly reduced scores for pain, swelling and stiffness of the biopsied joint postprocedure. We observed a median reduction of 39 mm in pain (V=45.5, p=4.04×10^−6^, 95% CI −50 to −27), a median reduction of 34 mm in stiffness (V=50.5, p=3.59×10^−6^, 95% CI −44.5 to −23.5) and a median reduction of 43 mm in swelling (V=32, p=2.34×10^−6^, 95% CI −54 to −31) (n=39 responses completed within 30 days of procedures, as one family did not complete symptom scoring; Wilcoxon paired t-test with p_adj_<0.016). This suggests that concomitant synovial tissue biopsy does not prevent the expected beneficial effects of intra-articular steroid injection^19^ (figure 2A).

Likert-scale tolerability questionnaires regarding the synovial tissue biopsy immediately after baseline procedures were answered with significantly positive responses (p_adj_<0.017 using Bonferroni’s post hoc test for three independent hypotheses for n=40 responses) (figure 2B–D). Participants reported symptomatic improvement in the biopsied joint compared with before the procedure (χ^2^=23.25, df=4, p=0.0001). Importantly, 80% of respondents said they would be ‘very likely’ or ‘somewhat likely’ to undergo the procedure again (χ^2^=59, df=4, p=4.7×10^−12^). Regarding pain, most participants reported ‘none’ or ‘mild discomfort’ (χ^2^=10.75. df=4, p=0.03; not significant with adjusted p). Positive responses were reported by participants across the age ranges (see online supplemental material for analysis of tolerability responses across age ranges). Consistent with this, the seven participants who underwent repeat biopsy procedures were from a diverse age range (coloured points, figure 1E, online supplemental table 4). No significant patterns of responses to post-procedure questionnaires were observed across different diagnostic groups (online supplemental figure 3).

No serious adverse events were reported following biopsy. Details of all adverse events and how they were classified can be found in online supplemental table 2 and 5 Online supplemental file 1. Synovial fluid leaking through the skin was observed in two participants post-procedure and one participant experienced prolonged superficial bleeding. Both of these adverse events have been previously reported following intra-articular steroid injection and, therefore, cannot be solely attributed to the biopsy procedure.^20^ No immediate flares of joint inflammation or post-procedural infections were observed.

### Synovial tissue biopsies in CYP provide tissue of a high quality that is suitable for research

At baseline, 59 formalin-fixed, paraffin-embedded synovial tissue samples were available for histological assessment by haematoxylin and eosin (H&E) staining. Across these samples, a median of 83% of tissue fragments were identified as synovium (IQR 69%–100%). Overall, 81.4% of samples (48/59) met predefined histological quality control (QC) criteria, requiring the presence of at least four tissue fragments, with ≥66.7% classified as synovium (QC scores for individual samples detailed in online supplemental table 6). These criteria were more stringent than those reported for adult studies due to the smaller relative volume of tissue obtained in our study.^21 22^ Synovial tissue obtained from participants exhibited preserved synovial tissue architecture, frequently including a discernible lining layer, which was hypertrophic in some samples, and sublining regions containing adipose tissue and infiltrating leukocytes (figure 3A). Three samples which did not pass histology QC also did not pass QC for single-cell RNA sequencing, suggesting histology QC is a useful measure of tissue quality. At 3/69 samples (4.3%), the overall proportion of poor-quality tissue samples was low in our study.

**Figure 3:**
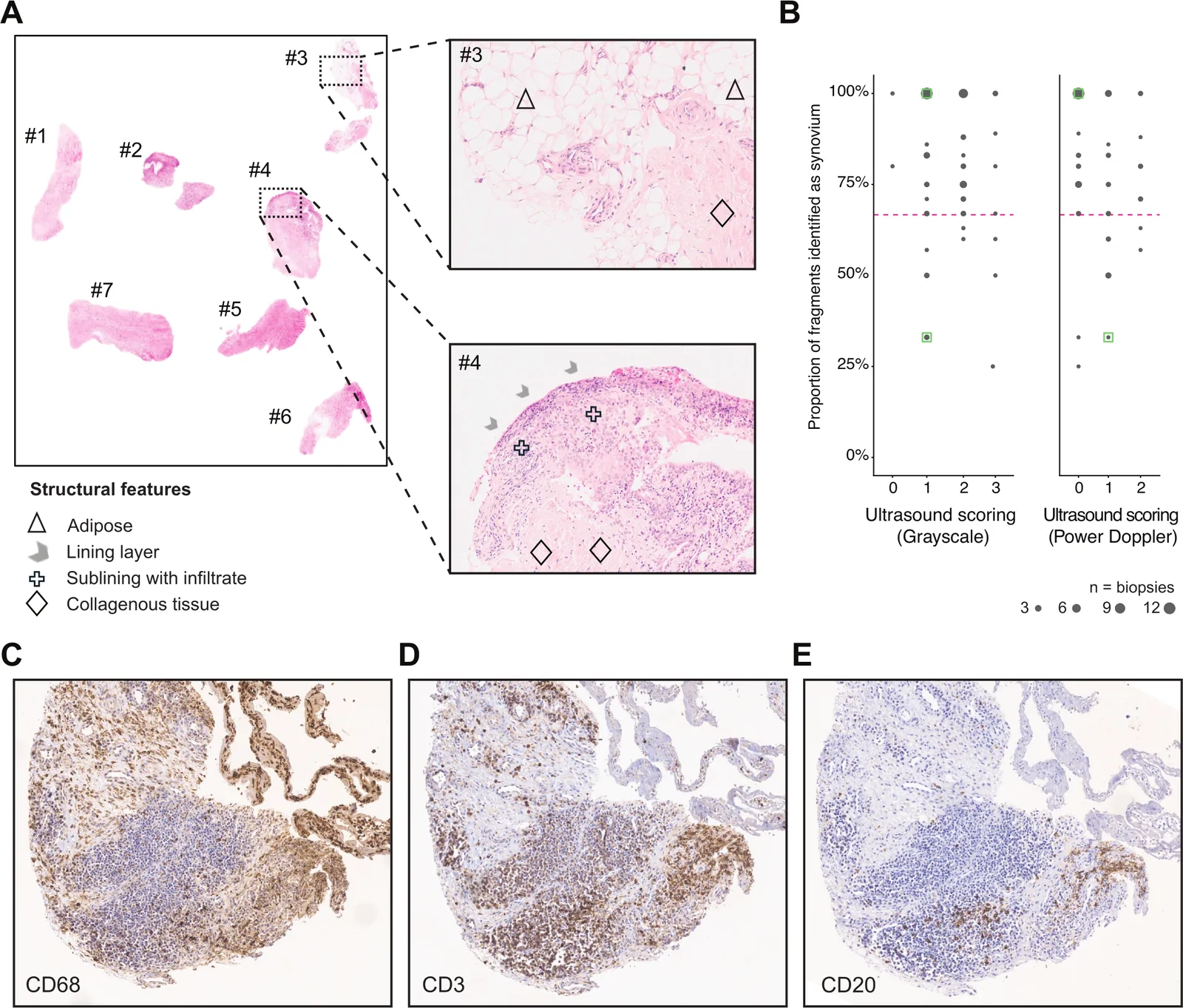
Synovial tissue biopsies from children provide high-quality tissue that is suitable for research. (**A**) Representative synovial tissue histology from a child with oligoarticular juvenile idiopathic arthritis (oJIA), with individual fragments numbered. Two fragments imaged at high magnification display distinct histological features, indicated by shapes. Magnification 0.6× (left) and 11.5× (right). (**B**) Count plots of ultrasound features recorded during biopsy procedure compared with the percentage of synovial tissue identified from histological assessment. Points in green squares show biopsies yielding fewer than four tissue fragments. Dashed line=66.7%. Kruskal-Wallis tests were performed across both greyscale (left) and power Doppler (PD) (right) compared with synovial tissue proportions (df=14 for both; greyscale: H=13.3, p=0.503, n=58 samples; PD: H=20.595, p=0.1125, n=53 samples). (**C-E**) Representative images of sequential paraffin-embedded synovial tissue specimens from a participant diagnosed with oJIA (magnification 9.5×), stained by immunohistochemistry for (**C**) CD68, (**D**) CD3 and (**E**) CD20.

To assess whether level of inflammation affected sample integrity, we examined how proportions of tissue fragments identified as synovial tissue related to ultrasonographic measures of arthritis severity (figure 3B). No significant association was observed between the overall proportion of synovial tissue acquired at biopsy and ultrasound grayscale or power Doppler scores (greyscale (n=58): p=0.503; power Doppler (n=53): p=0.1125, figure 3B). This demonstrates that high-quality synovial tissue could be obtained from joints with both high and low levels of inflammation (figure 3B). Furthermore, no association was observed between synovial tissue quality (as determined by % synovial tissue fragments >66.7%) and participant age or diagnosis (online supplemental figures 3 and 4). To examine cellular organisation within the tissue, serial tissue sections were stained for CD68, CD3 and CD20 by immunohistochemistry (figure 3C-E). This confirmed the expected pattern of synovial tissue architecture, with the synovial lining layer populated with CD68^+^ macrophages; CD3^+^ T clusters located in the sublining and CD20^+^ B cells localised in discrete tissue niches.

### Histological scoring of JIA synovial tissue reveals increased inflammatory scores in polyarticular RF-negative subtypes

A focused cohort of synovial tissue biopsy sections from 32 participants in which synovial lining could be observed, specifically including those diagnosed with oJIA and RF-pJIA, were scored for immune cell infiltration and lining layer hyperplasia (Krenn scoring,^23^ figure 4A–D). Histology and multiplexed immunofluorescence staining revealed marked structural and cellular distinctions in tissue fragments with high and low Krenn inflammatory scores (figure 4A-B). Between assessors, there was good interobserver agreement, suggesting this scoring approach can provide reproducible measures of inflammation in paediatric samples (Cohen’s weighted kappa estimate 0.62 (range 0.435–0.8), online supplemental figure 5). Higher Krenn scores were associated with increased inflammatory infiltrate and with an RF-pJIA diagnosis (Student’s t-test: p=0.029, 95% CI 0.08 to 1.27, figure 4B,C). Furthermore, in these 32 samples, the infiltrate component of the Krenn score significantly correlated with power Doppler scores at the time of the biopsy (Wilcoxon unpaired t-test: power Doppler score 0 vs 2: W=12.5, p=0.024, 95% CI 1 to 1.25; power Doppler score 1 vs 2: W=4, p=0.28, 95% CI 0.0 to 1.0). This illustrates that tissue pathology can be linked to the ultrasonographic observations of joint inflammation (figure 4D).

**Figure 4:**
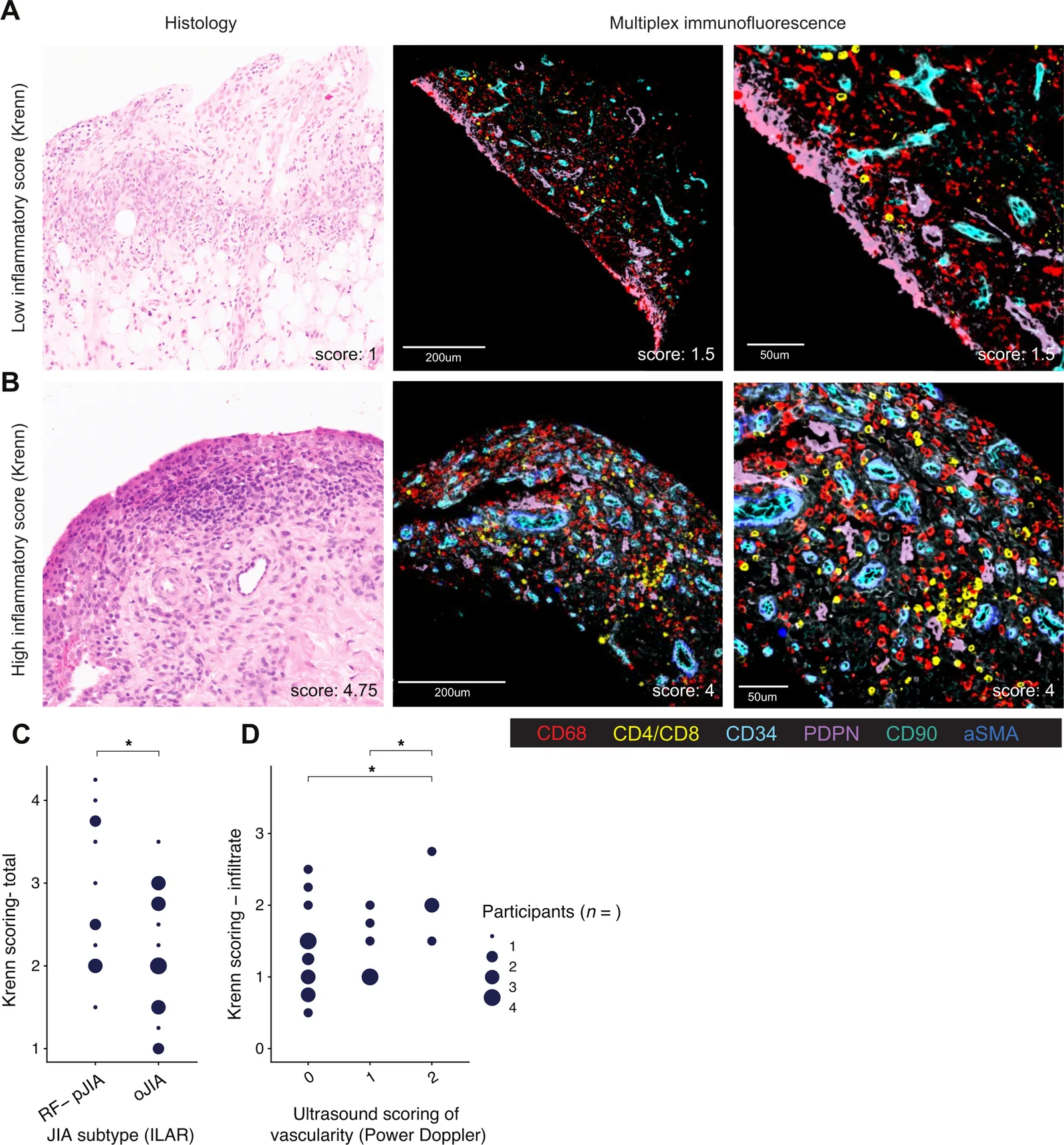
Histological scoring of synovial tissue from children with JIA. (**A-B**) Representative images of synovial tissue stained by H&E (left) and multiplexed immunofluorescence (middle, right) for samples with a low (**A**) and high (**B**) inflammatory Krenn score (20× magnification, scores indicated bottom right). Markers as per the colour key. (**C**) Count plot showing Krenn scores (infiltrate+lining layer) for participants diagnosed with RF-pJIA and oJIA (n=32 biopsies; unpaired t-test: p=0.029, 95% CI 0.076 to 1.275). (**D**) Count plot comparing PD ultrasound scores and the inflammatory component of the Krenn score (n=28 participants; 4=not recorded, Wilcoxon signed-rank unpaired t-test: PD score 0 vs 2: W=12.5, p=0.024, 95% CI 1.037 to 1.250; PD score 1 vs 2: W=4, p=0.028, 95% CI 0.000 to 1.000). *, p < 0.05. ILAR, International League of Associations for Rheumatology; JIA, juvenile idiopathic arthritis; oJIA, oligoarticular JIA; PD, power Doppler; RF-pJIA, rheumatoid factor-negative polyarticular JIA.

## Discussion

Synovial tissue analysis is urgently needed to advance our understanding of the pathobiology of childhood-onset inflammatory arthritis, permitting the exploration of, for example, tissue-based biomarkers of prognosis and treatment response in the early phases of the disease. Here, we demonstrate that minimally invasive, ultrasound-guided synovial tissue biopsies performed in CYP are safe, well-tolerated and acceptable to children, young people and their families. Our study illustrates that they can be performed even in very young children within a short timeframe from clinical diagnosis and as part of routine joint injection procedures performed under general anaesthetic during clinical care. Furthermore, biopsies taken during these procedures yield high-quality synovial tissue suitable for histological assessment and other downstream tissue analytics.

In CYP with JIA, synovial biopsies have been performed in a small number of studies for the purpose of research.^24–30^ Arthroscopy, synovectomy or joint replacement surgeries are also common sources of paediatric synovial tissue,^31–36^ with archived material also used,^37 38^ but these typically only capture synovial pathology in advanced stages of disease. Finnegan *et al* were some of the first investigators to employ synovial biopsies alongside intra-articular steroid injections,^24^ with analysis revealing distinct histological patterns of disease between different ILAR subtypes. However, more extensive large-scale efforts have been hampered in paediatric research by the continued lack of data pertaining to the safety and tolerability of procedures in this population. Here, we present the largest cohort, to date, of CYP with JIA undergoing synovial tissue biopsies and demonstrate the feasibility of embedding these procedures within routine clinical care pathways. We have recently performed synovial tissue analysis using single-cell profiling technologies to define the cellular heterogeneity of the inflamed JIA synovium.^13^ Our work, which used samples generated from this study workflow, demonstrates the incongruence of cellular composition between synovial tissue, synovial fluid and blood, highlighting the need for analysis of the target tissue in this disease.

We observed that synovial biopsy did not result in increased pain post-procedure and yielded the expected outcome from a steroid injection into the joint (reduced pain, stiffness and swelling).^19^ We observed no significant adverse effects following biopsy and a high degree of acceptability of the procedure, with a willingness to consent for a repeat biopsy in the future. In fact, seven participants underwent repeat biopsy procedures, with one participant having two further biopsies within the study window. A limitation of this study is the absence of a comparator group undergoing intra-articular steroid injection without synovial biopsy, precluding assessment of biopsy-specific tolerability. An adequately powered future study is planned to address this question.

Our observations are consistent with biopsy studies in adults, including a multicentre study of 524 synovial biopsies, which demonstrated that such procedures are well-tolerated and safe, even without concomitant steroid injection.^39^ Compared with adult procedures, participant-reported tolerability following the procedure was higher in our paediatric cohort.^39–41^ This difference may be attributed to the concomitant use of steroid injections in all but two procedures, as the addition of concomitant steroid has been shown to further improve tolerability in adult cohorts.^39^ It may also be attributed to the fact that the biopsy procedures we describe in our JIA cohort were performed under general anaesthetic, which was not the case for adult procedures. Further work must be carried out to demonstrate the tolerability of synovial tissue biopsies in CYP without general anaesthetic in appropriate older age groups. Furthermore, the majority of joints biopsied in this study were of the knee joint, and therefore future work will focus on the safety and tolerability of biopsies of smaller joints in paediatric cohorts.

Across studies of adults with arthritis, minimally invasive synovial biopsy procedures are performed using either needle or portal and forceps techniques, where higher yields of tissue per biopsy are typically reported than in the present study. This likely reflects both our exclusive use of a needle-based approach and the lower synovial tissue bulk associated with smaller joints in children compared with adults. Indeed, the weak correlation observed between number of tissue cores removed and the child’s age may reflect judgements made by the biopsy operator regarding the size of the joint in younger individuals. Despite this, we have demonstrated that this workflow generates tissue suitable for downstream research applications, including histology, single cell and spatial transcriptomics.^13^ We observed comparable synovial tissue quality by histological analysis of samples obtained from adults.^40^ Higher Krenn histological scoring was observed in RF-pJIA compared with oJIA; despite higher cJADAS10 scores in RF-pJIA participants, cJADAS10 and Krenn scores did not correlate, highlighting the potential of synovial biopsies to provide information regarding the local microenvironment, rather than systemic features of disease.

The cohort of CYP in the present study is young compared with previous studies of JIA synovial tissue, which is particularly pertinent to the investigation of ANA-positive oJIA, in which arthritis onset in children younger than 6 years of age is frequent, with no adult equivalent.^3^ We suggest that the acceptability of these procedures in young children will reduce hesitation in progressing tissue research for all subtypes of JIA across the age range. The feasibility of synovial biopsies in early disease (in our study, median 24.5 days from diagnosis to biopsy) has potential to advance investigation of JIA pathophysiology by circumventing the confounding variables of symptom duration and treatment effects encountered when sampling individuals with more established disease. Furthermore, our study population was representative of the UK as a whole, in terms of ethnic heritage.

While our study was open to all JIA subtypes, an important limitation is that recruitment was embedded within routine clinical care, resulting in an over-representation of oJIA. This reflects current clinical practice, in which children with oJIA are more likely to be referred for intra-articular corticosteroid injections as part of standard care. In contrast, systemic JIA and RF-positive polyarticular JIA were not represented in our cohort, likely because these patients are often treated promptly with systemic corticosteroids, DMARDs or biologic therapies without requiring joint injections.

To address this limitation, we are currently undertaking studies in which joint biopsies are performed under local anaesthesia with Entonox (nitrous oxide/oxygen). We anticipate that this approach will broaden participation to CYP who are not undergoing a clinically indicated procedure under general anaesthesia but are willing to undergo a research biopsy. This strategy may improve representation of subtypes underrepresented in this study, including systemic JIA and RF-positive polyarticular JIA, thereby reducing the selection bias towards oJIA inherent in the current study design.

Biopsy procedures in our study were performed by interventional radiologists, as our experience of synovial biopsies in adult cohorts demonstrated that ultrasound proficiency is important for their success. Capacity building to expand the application of ultrasound-guided minimally invasive biopsies in CYP with JIA is a key strategic objective of the TRICIA consortium (https://tricia.network/), through which we aim to provide training for standardised synovial biopsy procedures across UK paediatric rheumatology centres to support the delivery of synovial tissue research, including large multicentre studies.

In conclusion, we summarise our experience of performing minimally invasive synovial biopsy procedures early in disease in a large cohort of CYP with JIA, concomitant with intra-articular steroid injection while under general anaesthetic, using a standardised protocol in multiple centres. The tissue obtained from these procedures was of high quality and suitable for downstream research applications, including paraffin embedding for histological analysis, multiplex immunofluorescence and disaggregation for single cell RNA sequencing. Establishing the safety and tolerability of this methodology represents a key step in moving towards biopsy-driven, pathology-led clinical trials in JIA.

## Supplementary material

10.1136/rmdopen-2026-006817 online supplemental figure 1

10.1136/rmdopen-2026-006817 online supplemental figure 2

10.1136/rmdopen-2026-006817 online supplemental figure 3

10.1136/rmdopen-2026-006817 online supplemental figure 4

10.1136/rmdopen-2026-006817 online supplemental figure 5

10.1136/rmdopen-2026-006817 online supplemental file 1

## Data Availability

Data are available on reasonable request.
