# Supplementary material for: Minimally invasive, ultrasound-guided synovial tissue biopsies are well-tolerated, safe and feasible in children and young people with inflammatory arthritis
Source: RMD Open. 2026 Oct 14;12(4):e006817. doi: 10.1136/rmdopen-2026-006817 (PMC13691829; doi:10.1136/rmdopen-2026-006817)
Supplement: Supplementary file 3 [file rmdopen-12-4-s003.pdf]

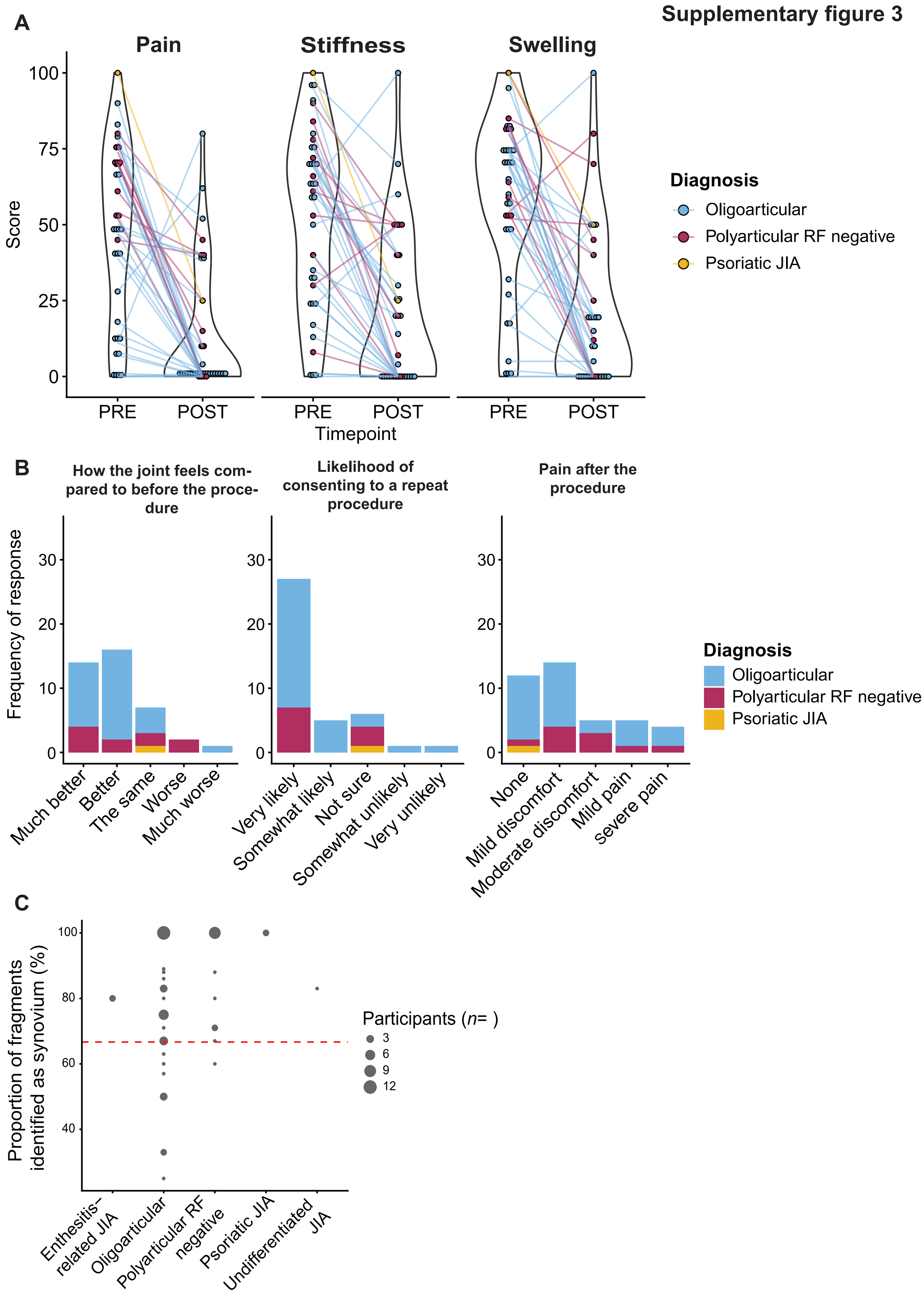
Supplementary figure 3
A
Pain
Stiffness
Swelling
Score
100
75
50
25
0
PRE
POST
Timepoint
Diagnosis
Oligoarticular
Polyarticular RF negative
Psoriatic JIA
B
How the joint feels compared to before the procedure
Likelihood of consenting to a repeat procedure
Pain after the procedure
Frequency of response
30
20
10
0
Much better
Better
The same
Worse
Much worse
Very likely
Somewhat likely
Not sure
Somewhat unlikely
Very unlikely
None
Mild discomfort
Moderate discomfort
Mild pain
severe pain
Diagnosis
Oligoarticular
Polyarticular RF negative
Psoriatic JIA
C
Proportion of fragments identified as synovium (%)
100
80
60
40
Enthesitis-related JIA
Oligoarticular
Polyarticular RF negative
Psoriatic JIA
Undifferentiated JIA
Participants (n=)
3
6
9
12
