# Supplementary material for: Minimally invasive, ultrasound-guided synovial tissue biopsies are well-tolerated, safe and feasible in children and young people with inflammatory arthritis
Source: RMD Open. 2026 Oct 14;12(4):e006817. doi: 10.1136/rmdopen-2026-006817 (PMC13691829; doi:10.1136/rmdopen-2026-006817)

Supplementary Figure 5

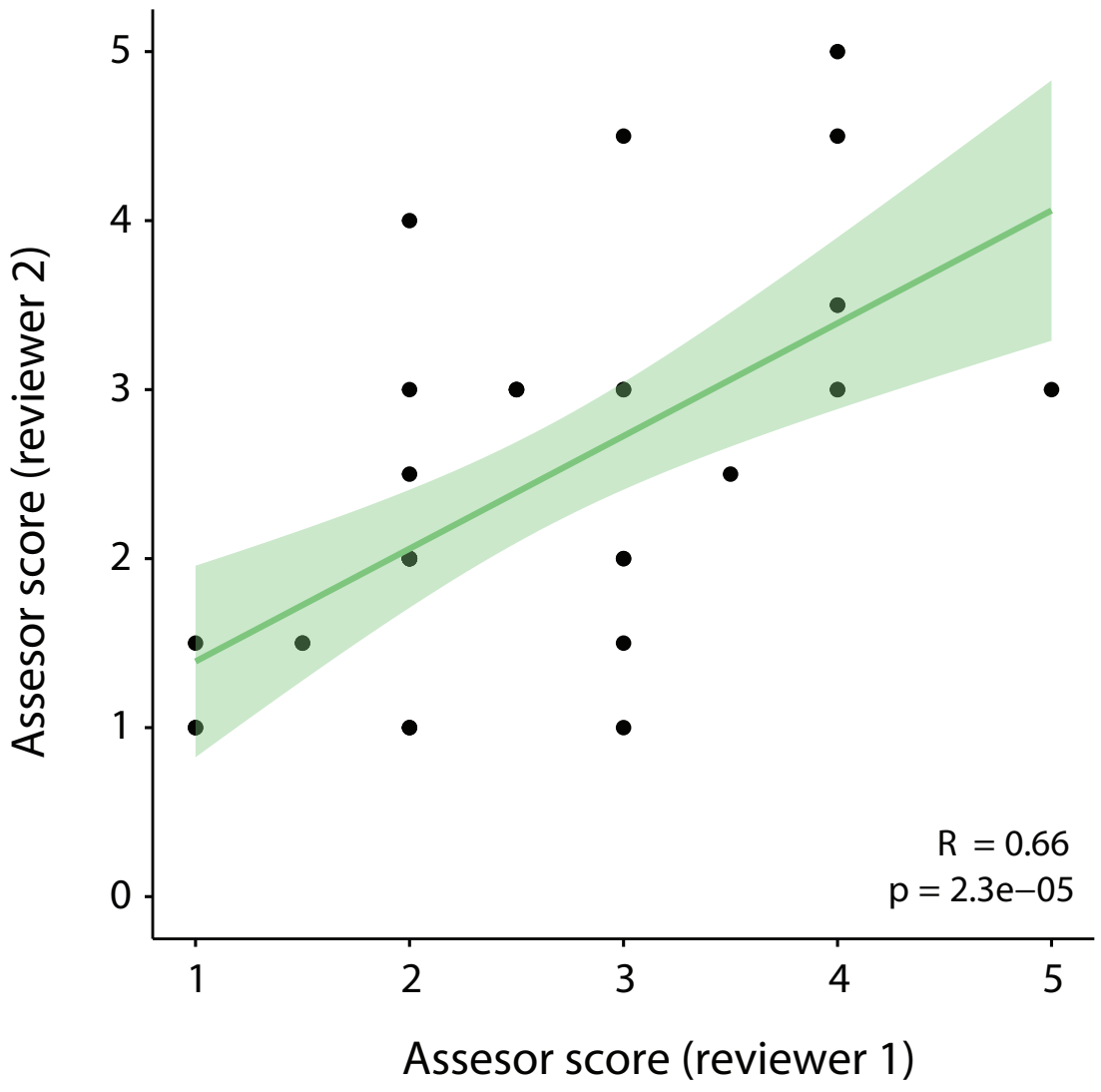

Supplement: Supplementary file 5 [file rmdopen-12-4-s005.pdf]
