# Supplementary material for: Minimally invasive, ultrasound-guided synovial tissue biopsies are well-tolerated, safe and feasible in children and young people with inflammatory arthritis
Source: RMD Open. 2026 Oct 14;12(4):e006817. doi: 10.1136/rmdopen-2026-006817 (PMC13691829; doi:10.1136/rmdopen-2026-006817)
Supplement: Supplementary file 6 [file rmdopen-12-4-s006.docx]

**Supplement: *Minimally invasive, ultrasound-guided synovial tissue biopsies are well tolerated, safe and feasible in children and young people with inflammatory arthritis***

**Supplementary acknowledgements**

***The Multiomic Analysis of Paediatric Joint and Gut inflammation (MAPJAG) study group*** Further to authors listed above (EC, CB, AH, CS, KK, IT, CF, CP, GN, CM, HA, HN, PC, SK, LJM, MT, ZS, PN, SC, SCL, FP, ECR, AF, EAA, LW, APC, the following are part of the MAPJAG study group who contributed to patient care, sample acquisition study design or data interpretation: Hussein Al-Mossawi, Rehana Begum, Ian Beh, Jenny Crook, Serena Cruickshank-Hull, Nia Evans, Lisa Fuller, Shamah Isaaq, Genevieve Gottschalk, Nadege Haouidji-Javaux, Ruth Howman, Neelam Khan, Kimme Hyrich, Ben Ingledow, Maryam Imran, Persephone Jenkins, Kelsey Jones, Saffron King, Brian Marsden, Diarmuid McLaughlin, Alyssia McNeece, Rafeeq Muhammed, Hannah Peckham, Nagesha Kendatti Muniyappa, Pauline Chan, Holm Uhlig, Qiong Wu, Emily Powell, Ovgu Kul Cinar and our patient and family partners, in particular Kerry Leslie and the CLUSTER Champions [CLUSTER Consortium Champions - CLUSTER Consortium](https://www.clusterconsortium.org.uk/cluster-consortium-champions/)

**Supplementary Figure legends**

**Supplementary Figure 1:** **Impact of participant age and ultrasound grading on the number of biopsy cores obtained from knee joints at baseline. (A)** Scatter plot showing age of the participant compared to the number of tissue cores removed; R = 0.34, p = 0.005 (Spearman’s Rank correlation coefficient). Regression line with 95% confidence interval shown in green. (**B**) Scatter plot showing ultrasound grading compared to the number of tissue cores removed. For **A** and **B**, baseline knee joint procedures (n = 65 knee joints, 58 participants) were assessed.

**Supplementary Figure 2:** **Comparisons between ‘Call Status’ groups**. (**A**) Age at biopsy procedure, (**B**) Time to biopsy from clinical diagnosis and (**C**) Number of active joints at baseline for patients: not responding to a post-procedure questionnaire (‘No Call’); responding after 30 days post-procedure (‘>30 days’); and those successfully completing post-procedure questionnaires within 30 days of their biopsy (‘<30 days’). Results from Kruskal-Wallis shown on each plot. Median and IQR shown per call status.

**Supplementary Figure 3:** **Tolerability of synovial biopsies across different diagnostic groups**. For (**A**) and (**B**); data as in figure 2, main text, but comparing by diagnosis subtype (ILAR category), coloured: blue = Oligoarticular; maroon = Polyarticular RF negative; yellow = Psoriatic JIA. Violin plots of questionnaire responses, completed before (PRE) and after (POST) the synovial biopsy procedure, using a Visual Analogue Scale (VAS). (**B**) Follow-up Likert-scale responses about the biopsied joint immediately after procedures and the next day. Question responses compared how the joint felt to before the procedure (left), likelihood of agreeing to a repeat procedure (middle) and pain after the procedure (left). (**C**) Count plots showing % of fragments deemed synovium per diagnosis group for all 59 samples available for histology assessment. Red dotted line at 66.67% shows acceptability cut-off. Kruskal-Wallis chi-squared = 13.41, df = 14, *p* = 0.49.

**Supplementary Figure 4: Impact of participant age on the proportion of synovium captured in biopsy procedures.** Scatter plot of synovial quality versus age at baseline procedures (n=59 samples, n= 52 participants); R = 0.14, p = 0.29 (Spearman’s rank correlation coefficient).

**Supplementary Figure 5: Intra-observer reliability of synovial histology scoring.** Scatter plot of Krenn scoring performed by two independent scorers for combined score of infiltrate and lining layer hyperplasia (n = 32 samples from oJIA and RF-pJIA); R=0.66, p=2.3x10^-5^ (Spearman’s rank correlation coefficient); Cohen’s weighted kappa estimate ~ 0.62 (lower 0.435 – upper 0.8).

**Supplementary Material and Methods**

**Synovial tissue histology and grading**

Paraffin-embedded synovial tissue was sectioned using a Leica 149MULTI0C1 microtome at 4 µm, sections were floated in a 30 °C water bath (Leica, HI1210) and placed onto a Leica X-tra adhesive slide (3800200AE). Slides were baked in an oven (CellPath, JAX-0400-00A) for one hour at 60 °C. Ten sections were taken: the first and seventh were stained with haematoxylin and eosin (H&E) for analysis at two different cutting levels. H&E staining was performed as follows. Slides were deparaffinised using Histo-Clear (Scientific Laboratory Suppliers, NAT1330) for five minutes twice, then 10 minutes once. For rehydration, slides were placed in 100% ethanol (VWR, 20821.330) for two minutes, then subsequently into 90%, 80%, 70% and 50% ethanol for two minutes each. To complete the rehydration process, slides were then placed into containers of distilled water three times for two minutes each.

Slides were then stained in filtered Harris haematoxylin solution (Pioneer Research Chemicals, PRC/R/51) for eight minutes, then washed in running tap water for five minutes before being placed in 1% acid alcohol (Pioneer Research Chemicals, PRC/R/75) for 30 seconds. Slides were then washed again in running tap water for one minute before being placed in 0.2% ammonia water (Sigma-Aldrich, 09859-250ML, diluted with distilled water) for bluing for a further minute. Slides were then washed again in running tap water for five minutes then rinsed by dipping 10 times in 95% ethanol. Slides were then counterstained in 1% aqueous eosin (Pioneer Research Chemicals, PRC/66/1) for five minutes before being dehydrated in a container of 95% ethanol for two minutes, followed by two changes of 100% ethanol for five minutes each. Slides were then placed in containers of Histo-Clear twice for five minutes before being mounted with DPX mountant (Sigma-Aldrich, 06522-500ML).

A repository of scanned H&E-stained sections was generated for the assessment and grading of synovial tissue (Leica Aperio eSLIDE Manager). Slides were scanned using a Leica Aperio AT2 brightfield scanner and digital magnification stated (0.2519 micron/pixel). Grading involved initially assessing all samples for synovial tissue quality (% tissue fragments deemed synovial tissue). For inclusion, samples required the presence of at least four tissue fragments and, additionally, 2/3rds of fragments had to be deemed as synovial tissue. Krenn scoring of infiltrate and lining layer, from a severity grading of 0-3 for each, was independently assessed by two pathologists per slide and a mean Krenn score obtained. Krenn scores for lining layer hyperplasia were included in the analysis only if both graders were in agreement that the slide had an adequate lining layer for scoring.

**Synovial tissue immunohistochemistry**

FFPE tissue sections were deparaffinised and rehydrated as described above and antigen retrieval performed at pH9 (Agilent Dako, S236784-2) for 20 minutes at 95˚C. Peroxidase block (Agilent Dako, S202386-2) was applied to slides followed by primary antibody (all Agilent Dako, CD68: M081401-2; CD3: A045201-2; CD20: M075501-2) in diluent (Agilent Dako, S202230-2). Polymer was applied followed by DAB to visualise antigen (Agilent Dako, K500711-2). Slides were counterstained with Harris haematoxylin solution (as above) and blueing buffer (Agilent Dako, CS70230-2). Slides were dehydrated and cleared, and mounted with DPX mountant (Sigma Aldrich, 06522).

**Immunofluorescence staining of synovial tissue (Leica CellDIVE)**

FFPE tissue sections were deparaffinised and rehydrated as described above and stained according to the CellDIVE protocol previously reported [10]. Antibodies used are listed in Supplementary Table 1, and where necessary conjugated using antibody labelling kits (Life Technologies:; A20187).

**Adverse Event Assessment**

Adverse events were assessed according to their severity as follows:

- *Mild: Symptoms noted but no disruption to normal activities of daily living (ADL)*
- *Moderate: Symptoms sufficient to disrupt normal ADL*
- *Severe: Symptoms sufficient to prevent normal ADL*

**Supplementary Results**

Further statistical analysis was performed on tolerability questionnaires assessing the influence of age and diagnosis on responses. An additional Chi-squared test was performed considering the different age categories (3x5 contingency table) (**Figure 2B**). There was a non-significant trend when adjusting for multiple comparisons for younger patients to report slightly more positively (3x5 contingency tables: comparing how the joint feels to before the procedure: _X_^2^ = 16.186, df = 8, p = 0.04; likelihood to consent to a repeat procedure: _X_^2^ = 16.53, df = 8, p = 0.04; pain/discomfort post procedure _X_^2^ = 10.895, df = 8, p = 0.2; *n* = 40 for each test, adjusted p*_adj_* < 0.017 using Bonferroni’s post-hoc test for three independent hypothesis).

The same statistics (3x5 contingency table Chi-squared test) were performed comparing diagnostic groups, with no significant patterns becoming apparent (**Supplementary Figure 3B**). Results were as follows: comparing how the joint feels to before the procedure: _X_^2^ = 12.852, df = 8, p = 0.117; likelihood to consent to a repeat procedure: _X_^2^ = 10.935, df = 8, p = 0.2054; pain/discomfort post procedure _X_^2^ = 7.2053, df = 8, p = 0.5147; *n* = 40 for each test.

**Supplementary Tables**

**Supplementary Table 1: Antibodies used for Cell DIVE multiplex imaging**

| Antibody | Clone | Source | Conjugation | In-house conjugation details |
| --- | --- | --- | --- | --- |
| Anti-podoplanin | D2-40 | Biolegend  916610 | Pre-conjugated |  |
| Anti-CD90/ Thy1 | EPR3132 | Abcam ab181885 | Conjugated in-house | AF555 |
| Anti-CD68 | EPR20545 | Abcam  ab280860 | Pre-conjugated |  |
| Anti-CD4 | EPR6855 | Abcam ab196372 | Pre-conjugated |  |
| Anti-CD8 | C8/144B | Biolegend  372906 | Pre-conjugated |  |
| Anti-CD34 | QBEND/10 | Abcam  Ab8536 | Pre-conjugated |  |
| Anti-alphaSMA | 1A4 | eBioscience/Thermofisher 53-9760-82 | Pre-conjugated |  |

**Supplementary Table 2. Classification of adverse events and causal relationships**

| ***Relationship*** | **Description** |
| --- | --- |
| ***Unrelated*** | There is no evidence of any causal relationship |
| ***Unlikely*** | Little evidence to suggest there is a causal relationship (lack of temporal co-relation) and another alternative more plausible explanation exists (co-morbidity, concurrent medications) |
| ***Possible*** | Evidence suggestive of a causal relationship (existence of temporal co-relation), but the involvement of other contributing factors cannot be excluded (co-morbidity, concurrent medications) |
| ***Probable*** | Sufficient evidence to suggest a causal relationship and the role of other factors is unlikely or minimal |
| ***Definitely*** | Clear causal relationship between intervention and adverse effect. Nil contribution by other factors. |
| ***Unknown*** | Insufficient evidence to assess for potential causal relationships. |

**Supplementary Table 3. Frequency of baseline diagnosis between post-procedure call status.** Diagnosis details for those who were not contactable, (‘No Call’), those that responded after 30 days, and those that responded within 30 days. Fisher test p=0.09

|  |  |  |  |  | Pain VAS | | Stiffness VAS | | Swelling VAS | | Post-procedure tolerability questionnaire | | |
| --- | --- | --- | --- | --- | --- | --- | --- | --- | --- | --- | --- | --- | --- |
| Patient number* | Diagnosis | Age category | Gender | Ethnicity | PRE | POST | PRE | POST | PRE | POST | How the joint feels in comparison to before the procedure | Likelihood of consenting to a repeat procedure | Pain after the procedure |
| 33 | Oligoarticular | 6-10yrs | F | Other | 14 | 2 | 31 | 0 | 58 | 10 | Much better | Very likely | None |
| 43 | Oligoarticular | 6-10yrs | F | White | 0 | 0 | 1 | 0 | 2 | 0 | The same | Somewhat likely | None |
| 44 | Oligoarticular | <6yrs | F | Asian | 83 | 0 | 97 | 70 | 65 | 50 | Better | Somewhat likely | Mild pain |
| 48 | Oligoarticular | <6yrs | F | White | 47 | 25 | 69 | 25 | 74 | 50 | Better | Somewhat likely | Moderate discomfort |
| 49 | Enthesitis-related JIA | 6-10yrs | M | White |  |  |  |  |  |  |  |  |  |
| 50 | Oligoarticular | <6yrs | M | White |  |  |  |  |  |  |  |  |  |
| 58 | Oligoarticular | >10yrs | M | White | 75 | 52 | 60 | 30 | 49 | 20 | Better | Not sure | Severe pain |
| * patient number equivalent to Supplementary Table 6 | | | | | | |  |  |  |  |  |  |  |

**Supplementary Table 4. Demographic and pre- and post-procedure questionnaire responses for patients who underwent repeat procedures**.

**Supplementary Table 5. Adverse Events reported in the study period.**

| **Details** | **Severity** | **Causal relationship** |
| --- | --- | --- |
| Leakage of fluid from needle puncture site | Mild | Possible |
| Superficial bleeding | Mild | Possible |
| Accidental needle puncture of skin during procedure | Mild | Definitely |
| Prolonged superficial bleeding | Moderate | Possible |
| Calf pain and swelling | Mild | Unlikely |
| Mild superficial haematoma | Mild | Possible |
| Leakage of fluid from needle puncture site | Mild | Possible |
| Skin and fat atrophy at site of injection | Mild | Possible |

**Supplementary Table 6. Details of repeat procedures, call status and histology in all 62 children/young people.** Totals per column at bottom in green.

| Patient Number | Joint(s) biopsied | Repeat: 2^nd^ Procedure | Repeat: 3^rd^ Procedure | Baseline Post Procedure Questionnaire Call Status | Baseline CS10/culture | Baseline Histology % synovium QC score if histology available. Highlighted if pass QC | Baseline Krenn analysis* |
| --- | --- | --- | --- | --- | --- | --- | --- |
| 1 | Left Knee |  |  | <30 | Yes | 88 | Yes |
| 2 | Right Wrist |  |  | <30 | Yes | Not available |  |
| 3 | Right Knee |  |  | <30 | Yes | 100 | Yes |
| 4 | Right Knee |  |  | <30 | Yes | 100 | Yes |
| 5 | Left Knee |  |  | <30 | Yes | 71 | Yes |
| 6 | Right Knee |  |  | <30 | Yes | 83 | Yes |
| 7 | Left Knee |  |  | >30 | Yes | 100 |  |
| 8 | Left Wrist |  |  | No Call | Yes | Not available |  |
| 9 | Left Knee |  |  | <30 | Yes | Not available |  |
| 10 | Left Knee |  |  | >30 | Yes | 100 | Yes |
| 11 | Left Knee |  |  | <30 | Yes | 83 |  |
| 12 | Right Knee |  |  | <30 | Yes | Not available |  |
| 13 | Left Knee |  |  | No Call | Yes | 100 | Yes |
| 14 | Left Knee |  |  | >30 | Yes | 100 | Yes |
| 15 | Right Knee |  |  | No Call | Yes | 86 | Yes |
| 16 | Left Knee |  |  | >30 | Yes | 60 |  |
| 17 | Right Knee |  |  | No Call | Yes | 100 | Yes |
| 18 | Left Knee |  |  | >30 | Yes | 83 |  |
| 19 | Right Ankle |  |  | >30 | Yes | 80 | Yes |
| 20 | Left Knee |  |  | >30 | Yes | 71 | Yes |
| 21 | Left Knee |  |  | No Call | Yes | 83 | Yes |
| 22 | Right Ankle |  |  | No Call | Yes | 100 | Yes |
| 23 | Right Knee |  |  | No Call | Yes | 67 | Yes |
| 24 | Left Knee |  |  | >30 | Yes | 63 |  |
| 25 | Right Knee |  |  | >30 | Yes | 100 | Yes |
| 26 | Right Knee |  |  | No Call | Yes | 100 | Yes |
| 27 | Right Knee |  |  | No Call | Yes | 100 | Yes |
| 28 | Left Knee |  |  | <30 | Yes | 50 |  |
| 29 | Right Knee |  |  | <30 | Yes | 100 |  |
| 30 | Left Knee |  |  | <30 | Yes | 100 | Yes |
| 31 | Left Knee |  |  | <30 | Yes | Not available |  |
| 32 | Right Knee |  |  | <30 | Yes | Not available |  |
| 33 | Right Knee | Yes |  | <30 | Yes | 67 | Yes |
| 34 | Right Knee |  |  | <30 | Yes | 100 |  |
|  | Left Knee |  |  |  | Yes | 100 |  |
| 35 | Right Knee |  |  | <30 | No | 100 <> |  |
| 36 | Right Knee |  |  | <30 | Yes | 33 |  |
| 37 | Right Knee |  |  | <30 | Yes | 71 | Yes |
| 38 | Left Knee |  |  | <30 | Yes | 100 | Yes |
| 39 | Right Knee |  |  | <30 | Yes | 100 | Yes |
| 40 | Left Knee |  |  | <30 | Yes | 88 | Yes |
| 41 | Right Knee |  |  | <30 | Yes | 67 | Yes |
| 42 | Left Knee |  |  | <30 | Yes | 100 |  |
| 43 | Right Knee | Yes |  | <30 | Yes | 89 |  |
|  | Left Knee |  |  |  | Yes | 75 |  |
| 44 | Right Knee | Yes |  | <30 | Yes | 75 | Yes |
| 45 | Right Knee |  |  | >30 | Yes | Not available |  |
| 46 | Right Knee |  |  | <30 | Yes | 67 | Yes |
| 47 | Right Knee |  |  | <30 | Yes | 60 |  |
| 48 | Left Knee | Yes |  | <30 | Yes | Not available |  |
| 49 | Right Knee | Yes |  | >30 | Yes | 80 |  |
|  | Left Knee |  |  |  | Yes | 80 |  |
| 50 | Right Knee | Yes | Yes | >30 | Yes | 67 |  |
|  | Left Knee | Yes | Yes |  | Yes | 100 |  |
| 51 | Right Knee |  |  | <30 | Yes | Not available |  |
| 52 | Right Knee |  |  | >30 | Yes | 75 |  |
|  | Left Lnee |  |  |  | Yes | 75 |  |
| 53 | Left Knee |  |  | <30 | Yes | 100 | Yes |
| 54 | Right Knee |  |  | <30 | Yes | 100 | Yes |
| 55 | Right Knee |  |  | <30 | Yes | 50 |  |
| 56 | Right Knee |  |  | <30 | Yes | 57 |  |
|  | Left Knee |  |  |  | Yes | 50 |  |
| 57 | Left Knee |  |  | <30 | Yes | 80 | Yes |
| 58 | Right Knee |  |  | <30 | Yes | 100 | Yes |
|  | Left Knee | Yes |  |  | Yes | 25 |  |
| 59 | Right Knee |  |  | <30 | Yes | 33 |  |
| 60 | Left Knee |  |  | <30 | Yes | 75 | Yes |
| 61 | Right Knee |  |  | <30 | Yes | Not available |  |
| 62 | Right Knee |  |  | <30 | Yes | 75 |  |
| **Total patients = 62** | **Total Baseline Samples = 69** | **Total 2^nd^ procedure = 8 samples from 7 patients** | **Total 3^rd^ procedure = 2 samples from1 patient** | **Total <30days = 40** | **Total samples CS10/ culture = 69** | **Total histology samples= 59 Total passing QC = 48/59** | **Total histology Krenn scored = 32** |
|  | **TOTAL SAMPLES = 79** | | |  |  |  |  |
|  |  | | |  |  |  |  |
| ^ Further QC pass involved: four fragments were available for assessment and >66.7% of these were deemed synovium | | | | | | | |
| *Krenn assessment performed when: tissue available for scorer; passing prior QC; adequate synovial lining layer within synovium; oligoarticular/polyarticular diagnosis | | | | | | | |
| <> Too few fragments to pass QC | | |  |  |  |  |  |
